# Redox regulation of cGMP-dependent protein kinase Iα in the cardiovascular system

**DOI:** 10.3389/fphar.2015.00139

**Published:** 2015-07-17

**Authors:** Oleksandra Prysyazhna, Philip Eaton

**Affiliations:** Cardiovascular Division, King’s College London, The British Heart Foundation Centre of Excellence, The Rayne Institute, St Thomas’ Hospital, London, UK

**Keywords:** protein kinase G, cGMP, disulfide dimerisation, oxidative modification, cardio-vascular system

## Abstract

Elevated levels of oxidants in biological systems have been historically referred to as “oxidative stress,” a choice of words that perhaps conveys an imbalanced view of reactive oxygen species in cells and tissues. The term stress suggests a harmful role, whereas a contemporary view is that oxidants are also crucial for the maintenance of homeostasis or adaptive signaling that can actually limit injury. This regulatory role for oxidants is achieved in part by them inducing oxidative post-translational modifications of proteins which may alter their function or interactions. Such mechanisms allow changes in cell oxidant levels to be coupled to regulated alterations in enzymatic function (i.e., signal transduction), which enables “redox signaling.” In this review we focus on the role of cGMP-dependent protein kinase (PKG) Ia disulfide dimerisation, an oxidative modification that is induced by oxidants that directly activates the enzyme, discussing how this impacts on the cardiovascular system. Additionally, how this oxidative activation of PKG may coordinate with or differ from classical activation of this kinase by cGMP is also considered.

## Introduction

Post-translational modification of proteins is a well-recognized mechanism of regulating their function. Such modifications include phosphorylation, glycosylation, acetylation, palmitoylation, sulfation, hydroxylation, proteolytic cleavage, as well as various oxidative modifications that are integrally involved in maintenance of homeostasis and adaptation. Of course these control mechanisms can become dysregulated during diseases, including those of the cardiovascular system. Perturbations in post-translational oxidative modification of proteins may be causal in the pathogenesis of such diseases.

Oxidation is a major class of protein post-translational modifications. These modifications result from reactions between protein amino acids and reactive oxygen species (ROS) or reactive nitrogen species (RNS). Methionine can be reversibly oxidized to the sulfoxide state by a range of ROS as well as irreversibly by forming a sulfone (Hoshi and Heinemann, 2001). Tyrosine can react with peroxynitrite to form 3-nitrotyrosine, with some evidence it is reversed by a denitrase enzyme (Irie et al., 2003). As the nitrotyrosine modification can potentially be reversed, this affords the theoretical prospect of reversible post-translational regulation. The amino acid cysteine contains a thiol (–SH) group on its side chain which in some proteins can react with oxidants to generate reversible modifications. Oxidants preferentially react with deprotonated (S^–^) thiols, referred to as thiolates. Most protein thiols are not in this ionized, thiolate state, and so do not commonly react with oxidants to form post-translational modification. However, some thiols are found in this reactive state and these are more readily susceptible to oxidative modification; this basal ionized state is a typical feature of many redox-active proteins. A protein thiolate (PS^–^) can react to form a number of different oxidation states such as sulfenic (PSOH), sulfinic (PSO_2_H) or sulfonic (PSO_3_H) acids, nitrosothiols (PSNO), as well as various disulfides (PSSR; Reddie and Carroll, 2008).

Disulfides can be formed within a protein (intradisulfide), between protein subunits (interdisulfide), with low molecular thiol-containing molecules such as glutathione (S-glutathionylation) or hydrogen sulfide (sulfhydration or sulfation; Rudyk and Eaton, 2014). Reversible protein modifications such as disulfide formation can be analogous to well-established post-translational modifications such as phosphorylation (Schroder and Eaton, 2008). Disulfide bond formation in proteins is a widely recognized cysteine modification. It can influence catalytic activity (Cremers and Jakob, 2013), protein—protein interactions (Banky et al., 2003) and subcellular localization (Brennan et al., 2006). Of course disulfide bonds also play crucial roles in maintaining the structural integrity and correct folding of many proteins (Betz, 1993). Redox proteomic studies searching for proteins that form disulfide bond have shown that a multitude of proteins involved in wide-ranging biological processes including redox homeostasis, chaperone activity, metabolism, transcriptional regulation, and protein translation (Leichert et al., 2008; Reddie and Carroll, 2008; Paulsen and Carroll, 2010) are potentially regulated in this way. When disulfides form in signaling proteins such as phosphatases or kinases, this allows changes in redox state to be integrated with regulation involving protein phosphorylation.

A broad range of protein phosphatases can be regulated by modulation of their thiol redox state, such as low molecular weight protein tyrosine phosphatase (LMW-PTP), phosphatase and tensin homolog (PTEN), cell division cycle dual-specificity phosphatase (Cdc25), protein tyrosine phosphatase 1B (PTP1B), protein tyrosine phosphatase 2α (PTP2α), Src homology region two domain-containing phosphatase-1 and -2 (SHP-1/2), (Salmeen and Barford, 2005; Chen et al., 2009; Paulsen and Carroll, 2010; Tanner et al., 2011). In the case of PTP, the reason disulfide or other modes of oxidation are inhibitory to their activity is because they have a catalytic thiolate that is integral to the dephosphorylation. This is because the thiolate enables a nucleophilic attack on its phospho-tyrosine substrates (Tonks, 2006). A number of kinases have also been shown to be redox regulated, including stress-activated MAPK/thioredoxin peroxidase 1(Sty1/Tpx1), Src tyrosine kinase, apoptosis signal-regulated kinase-1 (ASK1; Paulsen and Carroll, 2010). Such redox regulation can also involve disulfide bond formation as occurs with protein kinase A RIα (Brennan et al., 2006), and the focus of this review, namely cGMP-dependent protein kinase—also known as protein kinase G (PKG; Burgoyne et al., 2007).

## PKG—isoforms, Structure, and Activation

**FIGURE 1:**
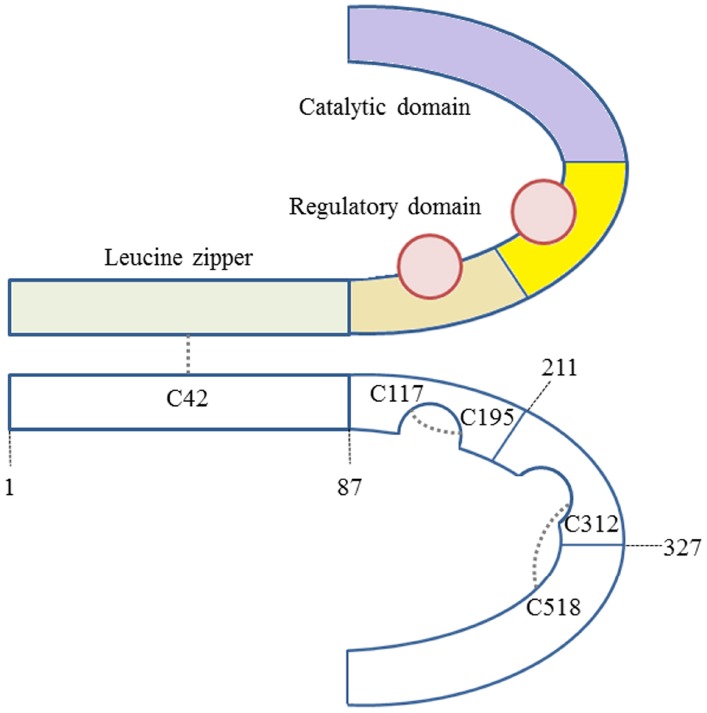
**PKG Iα contains three functional domains—an N-terminal leucine zipper, a regulatory and a catalytic.** There are three pairs of cysteines, which may form disulfide bridges: C117-C195, C312-C518, and C42-C42.

PKG is a member of the serine/threonine kinase family. Mammals have two PKG genes, *prkg1* and *prkg2*, that encode PKG type I and type II, respectively. PKG I and PKG II are homodimers of two identical subunits (≈75 or ≈85 kDa, respectively) and have similar domain architecture. PKG contains three functional domains (Francis et al., 2010; Figure 1).

The N-terminal domain contains an α-helix with a hydrophobic leucine/isoleucine zipper motif that is responsible for the basal dimerisation of the kinase and its interaction with so-called G-kinase anchoring proteins (GKAPs; Scholten et al., 2008). The regulatory domain on each subunit contains one high affinity and one low affinity cyclic guanosine monophosphate (cGMP) binding site. The catalytic domain, consisting of an ATP/Mg^2+^ and a substrate binding site, catalyzes the transfer of the γ phosphate of ATP to the hydroxyl group of a serine/threonine side chain of the target protein. Binding of cGMP to both cGMP binding domains induces a conformational change that relieves the inhibition of the catalytic core by the N-terminus and allows the phosphorylation of substrate proteins (Feil et al., 2003; Scholten et al., 2008). The N-terminus (approximately amino acids 1–100) of PKG I is encoded by two alternatively used exons resulting in the production of two PKG I isoforms, namely PKG Iα and PKG Iβ. Although PKG Iα and Iβ do not differ much in sequence beyond the N-terminus, PKG Iα has more than 10-fold higher affinity for cGMP than PKG Iβ (Ruth et al., 1991; Lee et al., 2011).

PKG isoforms also differ in their tissue and cellular distributions. PKG I is predominantly localized in the cytoplasm, whereas PKG II is typically anchored to the plasma membrane by N-terminal myristoylation (Vaandrager et al., 1996). The PKG Iα isozyme is mainly found in lung, heart, dorsal root ganglia, and cerebellum. In contrast, the Iβ isozyme is highly expressed in platelets, as well as hippocampal and olfactory bulb neurons. Smooth muscle cells, for example, within the uterus, blood vessels, intestine or the trachea, contain both Iα and Iβ isozymes (Geiselhoringer et al., 2004). PKG II was found mainly in kidney, cerebellum and mucosa (Jarchau et al., 1994).

PKG Iα contains 11 cysteine residues (Takio et al., 1984), five of which have been suggested to contribute to oxidation-induced activation (Figure 1). Oxidant-induced PKG Iα disulfide bond formation and activation was proposed as a complimentary mechanism to cyclic nucleotide-mediated regulation of kinase activity. Oxidants induce interprotein disulfide bond formation between C42 on each of the adjacent chains in the PKG Iα homodimer complex, rendering the kinase catalytically active independently of cGMP (Burgoyne et al., 2007). Two potential intradisulfide bonds have also been reported to form within PKG Iα in response to metal ion-induced oxidative stress. It was likely that one interdisulfide forms between C117 and C195 and the other between C321 and C516, although it was unclear if both can be present simultaneously within the same monomeric chain (Landgraf et al., 1991; Osborne et al., 2011). The redox state of C42 mediating targeting of PKG Iα would appear rationale as it is within the established leucine zipper motif that mediates its interaction with substrates (Michael et al., 2008; Scholten et al., 2008). Perhaps C117-C195 disulfide, which is within a cGMP binding domain and was present within crystallized PKG (Osborne et al., 2011), is a more logical candidate for mediating catalytic competence induced by oxidants. Although it is also notable that C312, which is located within the other cGMP binding pocket, can also form interdisulfide bonds. One possibility is that oxidants induce separate targeting and activating disulfides, with genetic or pharmacological interventions that prevent either of their formation limiting PKG signaling responses to oxidants. Studies with metal ion-induced oxidation identified the intraprotein disulfides, whilst those with H_2_O_2_ (Landgraf et al., 1991; Osborne et al., 2011), nitrosocysteine (Burgoyne and Eaton, 2009), or H_2_S (Landgraf et al., 1991; Osborne et al., 2011) identified the interprotein disulfide. It is possible that each of these oxidants simultaneously induced all of the several disulfides that can form, but they were not reported in some studies because they could not be determined or were not specifically assessed. Conceptually it is possible that there is selectivity in the precise modification different oxidants induce. This is due to different oxidants having distinctive physicochemical properties, together with individual protein thiols having disparate reactivity and solvent accessibility due to their specific environments. Further work would be required to define if a specific ROS species can indeed selectively induce a particular disulfide bond in PKG.

It is evident that PKG Iα can be activated by cGMP binding to the kinase (“classical activation”) or alternatively by disulfide bond formation (“oxidant activation”), although the two mechanisms may have some positive cooperativity (Dou et al., 2012). However, cGMP binding to PKG Iα promotes resistance to C42 interprotein disulfide bond formation. Accordingly, cGMP depletion sensitizes PKG Iα to oxidation (Burgoyne et al., 2012). Similarly, a cGMP mimetic attenuated H_2_O_2_-induced kinase interprotein disulfide formation (Muller et al., 2012). In contrast, pre-oxidation of the kinase with H_2_O_2_ slightly impaired its activation by cGMP (Muller et al., 2012).

## The Role of PKG Iα Disulfide Dimerisation in Blood Vessels

**FIGURE 2:**
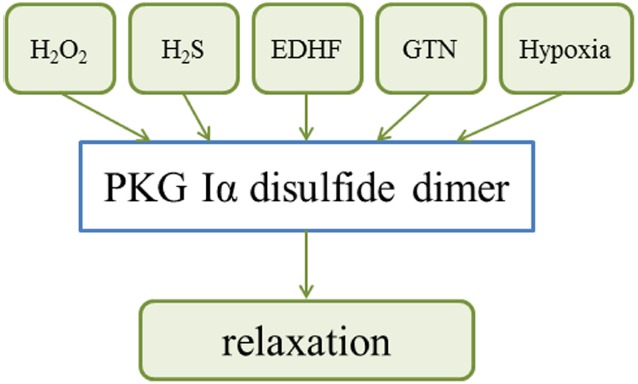
**An overview of how PKG Iα disulfide dimerisation integrates a variety of stimuli to induce vasodilation and blood pressure lowering**.

The oxidative activation of PKG Iα by C42 interprotein disulfide formation is an important mechanism contributing to blood pressure homeostasis, being a component of endothelium-derived hyperpolarizing factor (EDHF)-dependent vasodilation (Figure 2). This regulatory mechanism was explored using a knock-in (KI) mouse expressing only a C42S “redox-dead” version of PKG Iα which is unable to form the active disulfide dimer. This subtle, single atom substitution abrogated the vasodilatory action of H_2_O_2_ on resistance vessels and resulted in hypertension *in vivo* (Prysyazhna et al., 2012b). Such oxidative activation of PKG Iα decreases vascular smooth muscle cell Ca^2+^, a mechanism that likely contributes to vasodilation induced by oxidants (Muller et al., 2012). PKG Iα C42 interdisulfide activation has been rationalized as an end-effector of EDHF-dependent blood pressure lowering that is mediated by H_2_O_2_ derived from uncoupled NOS (Shimokawa, 2014). This mechanism also contributed to human coronary arteriole vasodilation mediated by oxidant-activated PKG opening of smooth muscle voltage and *Ca*^2+^ activated potassium (BK) channels (Zhang et al., 2012). Activation of these plasma membrane proteins is consistent with H_2_O_2_ promoting the translocation of PKG Iα from the cytoplasm to the membrane in porcine coronary arteries. This event was associated with potentiated PKG activity as well as vasodilation of porcine coronary arteries to a nitric oxide donor or 8-Br-cGMP (Dou et al., 2012). This contrasts evidence that PKG Iα oxidation leads to its impaired activation by cGMP in embryonic fibroblasts or vascular smooth muscle cells (Muller et al., 2012), although these differences may simply be due to the different models studied. Disulfide activation of PKG Iα by dimerization mediates relaxation of bovine coronary arteries to hypoxia, which was also associated with oxidation of cytosolic NADPH and phosphorylation of the PKG substrate protein vasodilator-stimulated phosphoprotein (VASP; Neo et al., 2011; Figure 2). Although these data support PKG Iα oxidation as a mechanism of EDHF-dependent vasodilation, it is notable that the evidence for other factors such as epoxyeicosatrienoic acids (EETs) being a principal mediator is especially robust (Fromel and Fleming, 2015). Although it is interesting to note that the epoxide moiety present in EETs can have thiol reactivity, leading to the idea that these lipid species could potentially react with C42 of PKG Iα to activate it.

**FIGURE 3:**
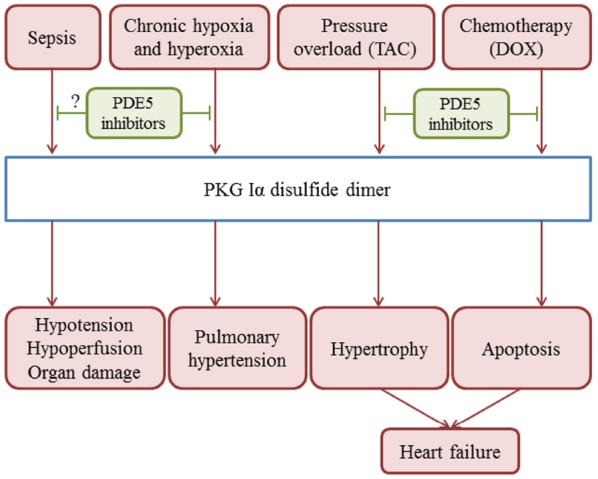
**Overview of the roles of PKG Iα disulfide dimerisation in the development of pathologies in the cardiovascular system**.

Interestingly, PKG Iα disulfide dimerisation also in part mediates H_2_S-induced blood pressure lowering, consistent with this established vasodilator being implicated as an EDHF and coupling to the opening of potassium channels (Zhao et al., 2001; Mustafa et al., 2011). Induction of PKG Iα in response to H_2_S is perhaps counterintuitive, as this molecule is an electron donor that is fully anticipated and capable of reducing disulfide bonds. Oxidation was rationalized by the demonstration that in the presence of oxygen or other oxidants, H_2_S rapidly forms polysulfides, which promote the oxidation of PKG via thiol-disulfide exchange reactions (Stubbert et al., 2014; Figure 2). This rapid oxidation of H_2_S is explained by the fact that at physiological pH, it principally exists in the oxidant-reactive deprotonated thiolate state considered above. PKG Iα disulfide formation also significantly mediates vasodilation and blood pressure-lowering induced by the commonly use drug nitroglycerin (GTN). GTN is metabolized to generate several reaction products, including some with thiol-oxidation capability. A redox-dead C42S PKG Iα KI mouse had markedly impaired blood pressure reduction following GTN treatment, (Rudyk et al., 2012) pointing to its important role for this mechanism *in vivo* (Figure 2). Over-activation of PKG Iα by disulfide induction occurs during sepsis in mice. Consistent with this, C42S PKG Iα KI mice are resistant to the hypotension and organ injury associated with sepsis (Rudyk et al., 2013; Figure 3).

Vascular smooth muscle cells of blood vessels in the airways are abundant in PKG Iα, with disulfide-activation mediating their responses to H_2_O_2_, hypoxia, hyperoxia, and some drugs used in the treatment pulmonary hypertension (Neo et al., 2010)., Both cGMP-dependent and a disulfide-dependent activation of PKG appear to contribute to hypoxic vasoconstriction in bovine pulmonary arteries (Neo et al., 2011; Figure 2). During hypoxia there is an increase in cellular reducing equivalents that couple to a reduction in disulfide-active PKG Iα. Loss of this vasodilatory mechanism leads to constriction of the pulmonary vessel (Neo et al., 2013). PKG Iα disulfide activation is a major contributing factor to the vasodilator actions of dehydroepiandrosterone, a steroid hormone with pulmonary vasodilator activity used to treat pulmonary hypertension. Dehydroepiandrosterone or hypoxia may each inhibit glucose-6-phosphate dehydrogenase, promoting NADPH oxidation and PKG disulfide activation. These vasodilatory responses were deficient in pulmonary arterial vessels from a C42S PKG Iα KI mouse (Patel et al., 2014). Disulfide-activation of PKG Iα is involved in the development of hyperoxia-induced lung injury (Figure 3). Compared to wild-type controls, C42S PKG Iα KI mice were protected from right ventricular hypertrophy, vascular remodeling and decreased vascularization associated with chronic hyperoxia (Lee et al., 2014; Figure 3). We conclude, drugs that modulate the redox-controlled activity of PKG activity may be of therapeutic value in the setting of airway diseases, such as chronic pulmonary hypertension or associated pathologies such as bronchopulmonary dysplasia.

## The Role of PKG Iα Disulfide Dimerisation in the Heart

Classical activation of PKG Iα is known to play an important role in the regulation of cardiac function in physiological and pathophysiological conditions. In contrast, a lot less is known about the impact of redox regulation of this kinase on the myocardium, especially compared to our understanding of its role in the vascular system, as considered above.

Classical cGMP-dependent activation of PKG is well known to regulate cardiac contractile function (Shah et al., 1995). PKG Iα disulfide-activation also appears important for the maintenance of myocardial relaxation. For example, diastolic dysfunction was observed by echocardiography in C42S PKG KI mice. Hearts from KI mice had a reduced diastolic volume, which could be an indicator of impaired relaxation (Prysyazhna et al., 2012b). Furthermore, a decreased ratio of the early (E) to late (A) ventricular filling velocities (E/A ratio), indexed by pulse wave Doppler analysis of mitral inflow velocity, in C42S PKG KI mice suggests their myocardium is stiffer and cannot relax with the same speed and efficiency as those of wild-type mice (Prysyazhna et al., 2012a). Additionally, studies in isolated, perfused hearts showed that hypoxia- or ischemia-induced elevations in end diastolic pressure were exacerbated in C42S PKG compared to wild-type controls. Thus, PKG Iα disulfide dimerisation is important for maintaining diastolic relaxation basally and during myocardial hypoxia or ischemia (Prysyazhna et al., 2012a).

The role of PKG Iα disulfide dimerisation in the development of heart failure was investigated using trans-aortic constriction (TAC), which significantly increases after-load and results in cardiac hypertrophy and failure. The C42S PKG KI mice were protected from TAC-induced hypertrophy compared to wild-types. Oxidized PKG Iα was largely located in the cytosol whereas classically-activated and non-oxidisable C42S PKG Iα translocated to the plasma-membrane where it suppressed transient receptor potential channel-6 to block adverse signaling during TAC (Nakamura et al., 2015; Figure 3). Like TAC, the widely used chemotherapy agent doxorubicin induces oxidative stress, and is associated with apoptotic cell death and decreased heart contractility. These maladaptive events induced by doxorubicin were significantly mediated by PKG Iα disulfide-activation as the redox dead C42S KI mice were resistant to the toxic effects of chemotherapy observed in wild-type controls. Loss of otherwise cardioprotective RhoA Ser188 phosphorylation when PKG Iα is oxidized was identified as a mechanistic link between interprotein disulfide formation in the kinase and apoptosis (Prysyazhna et al., 2013). Thus, PKG Iα disulfide activation in the myocardium appears maladaptive during scenarios that induce oxidative stress, such as TAC or doxorubicin chemotherapy (Figure 3). These observations may explain why there was a lack of hypertrophy or heart failure in the C42S PKG Iα transgenics despite them living chronically with significant hypertension (Prysyazhna et al., 2012b). We conclude that therapies that prevent this PKG Iα disulfide formation may have therapeutic value. The oxidant-activation of PKG Iα is not restricted to smooth muscle cells or cardiomyocytes. For example, PKG Iα underwent interprotein disulfide bond formation in response to exogenous H_2_O_2_ in cultured rat podocytes. This, potentially causatively, induced changes in the actin cytoskeleton organization and increased albumin permeability across the podocyte filtration barrier. Thus, redox modulation of PKG Iα may regulate renal filtration (Piwkowska et al., 2012).

**FIGURE 4:**
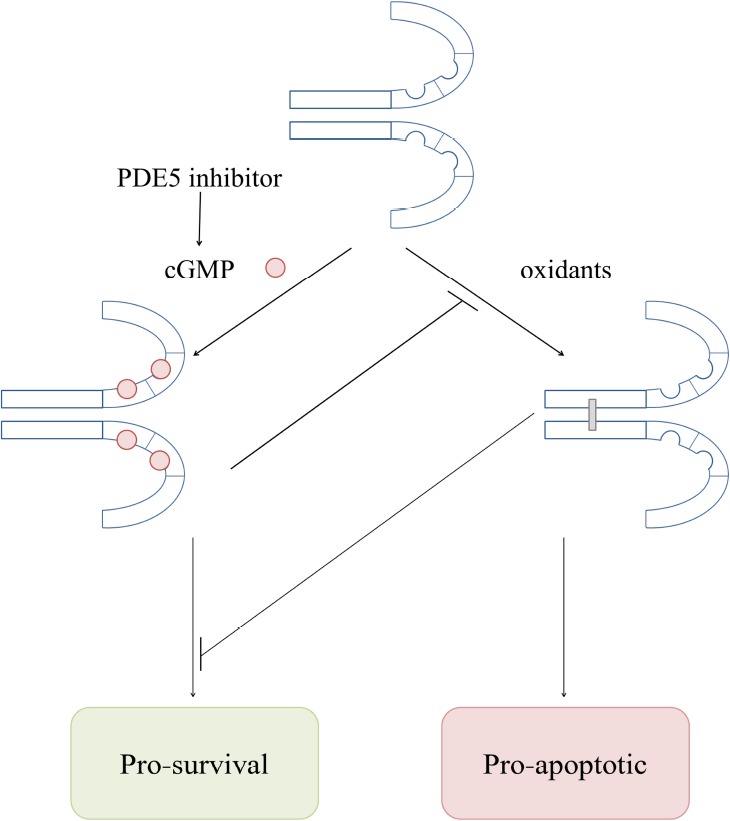
**Overview of the complex interaction between cGMP- and oxidant-induced PKG Iα activation mechanisms in the heart**.

The NO-cGMP-PKG pathway is known to be anti-apoptotic (Kim et al., 1999; Fiscus, 2002; Fiscus et al., 2002). PDE5 inhibitors (for example Sildenafil or Tadalafil), which increase cGMP levels, are protective against heart failure in different animal models and also in humans (Fisher et al., 2005; Takimoto et al., 2005; Nagayama et al., 2009; Guazzi et al., 2011; Blanton et al., 2012). The observation that cGMP blocks PKG oxidation was made in two independent studies (Burgoyne et al., 2012; Muller et al., 2012), leading to the hypothesis that the protective effects of PDE5 inhibitors could be explained by cGMP-elevation limiting PKG Iα oxidation. That would suggest that PKG disulfide bond formation in the myocardium is maladaptive, leading to apoptosis and heart failure (Figure 4).

The PDE5 inhibitor Vardenafil was found to be protective against noise-induced hearing loss through a cGMP/PKG-dependent increase of poly ADP ribose polymerase, a protein which promotes DNA repair, activity. Stimulation of this mechanism prevented noise-induced hair-cell dysfunction and cell death. Mice with deletion of PKG I were found to have a higher vulnerability to noise-induced hearing loss and were not protected by PDE5 inhibition, consistent with PKG I mediating protection (Jaumann et al., 2012).

The C42 residue within PKG Iα that can form the interprotein disulfide is located within the N-terminal leucine zipper, which is known to be responsible for the kinase targeting to substrates such as myosin phosphatase (Surks et al., 1999). It is rationale to suggest that alterations in this important targeting domain, such as disulfide formation, may modulate the interaction of the kinase with GKAPs or substrates. However, this possibility remains largely unproven.

The role of the leucine zipper in PKG binding to RhoA was demonstrated by studies in which subtle alterations to the kinase amphipathic helix prevented their binding. Thus phosphorylation and inactivation of RhoA requires cGMP-activated PKG Iα with an intact leucine zipper (Kato et al., 2012). Several studies have demonstrated the importance of the N-terminal leucine zipper targeting domain for correct kinase function through the use of leucine zipper mutant (LZM) mice. These LZM transgenics, engineered to have a mutation in the PKG Iα N-terminal domain to prevent it binding to targets like the myosin-binding subunit of myosin phosphatase (Surks et al., 1999), displayed vascular smooth muscle cell abnormalities, impaired vasorelaxation and increased systemic blood pressure. This was at least in part due to impairment of PKG Iα-mediated RhoA/Rho kinase inhibition (Michael et al., 2008). The same LZM mice had enhanced RhoA-GTPase activity in their lungs, which resulted in pulmonary constriction and a consequential progressive increase in right ventricular systolic pressure and right heart hypertrophy during normoxia. These adverse events due to pulmonary hypertension were exacerbated by chronic hypoxia compared to wild-type controls, and could not be corrected by the PDE5 inhibitor Tadalafil (Ramchandran et al., 2014). These LZM mice also had potentiated pathologic cardiac hypertrophic responses to pressure overload. Furthermore, the zipper mutant mice lacked the Sildenafil-mediated protection from TAC afforded to wild-type controls (Blanton et al., 2012).

We conclude that PKG Iα disulfide dimerisation is an important regulatory mechanism, involved in the maintenance of health (e.g., blood pressure regulation, diastolic relaxation, kidney filtration), but these processes can be dysregulated to causatively contribute to cardiovascular pathologies (e.g., sepsis, hypoxia, heart failure). Drugs that modulate the redox state of PKG Iα may have therapeutic potential.

### Conflict of Interest Statement

The authors declare that the research was conducted in the absence of any commercial or financial relationships that could be construed as a potential conflict of interest.
